# Corrigendum to: Sex-specific differences in swimming, aerobic metabolism and recovery from exercise in adult coho salmon (*Oncorhynchus kisutch*) across ecologically relevant temperatures

**DOI:** 10.1093/conphys/coab100

**Published:** 2022-01-11

**Authors:** K Kraskura, E A Hardison, A G Little, T Dressler, T S Prystay, B Hendriks, A P Farrell, S J Cooke, D A Patterson, S G Hinch, E J Eliason

*Conserv Physiol* 9(1): coab016; doi: 10.1093/conphys/coab016

Due to an error with production, when this paper was first published, it contained an incorrect supplementary data file from another paper. This has now been corrected online, and the correct supplementary data is now accessible with this paper online. The publisher apologizes for this error. The paper was also originally missing a data availability statement, which should have read: ``Supplementary material is available at Conservation Physiology online. Data are available on Dryad, DOI: https://doi.org/10.25349/D9390K''. This has been corrected, and the data availability statement is now available in the published paper online.

